# Intramural Jejunal Hematoma Causing Intermittent Bowel Obstruction—A Rare Manifestation of Warfarin Toxicity

**DOI:** 10.1055/s-0042-1755223

**Published:** 2022-11-06

**Authors:** Arun S. Patil, Shriya Shriya, Nikhil Dhimole, Jalbaji More

**Affiliations:** 1 Department of General Surgery, Grant Government Medical College, Sir JJ Group of Hospitals, Mumbai, Maharashtra, India; 2 Consultant Laparoscopic Surgery, Vishwa Hospital, Nanded, Maharashtra, India

**Keywords:** warfarin toxicity, intramural jejunal hematoma, intestinal obstruction

## Abstract

Patients with thromboembolic disorders are commonly on anticoagulants; hence, they are susceptible to bleeding episodes such as ecchymosis, gingival, subconjunctival bleeding, and rarely can have intramural hematoma of small bowel causing patient to present with intestinal obstruction. It is a rare cause of mechanical bowel obstruction requiring a nonsurgical management.

Our patient was a 55-year-old male, a known case of thromboembolism on warfarin medication, presented with abdominal pain and vomiting. Patient's laboratory reports reflected anemia and deranged coagulation profile with prothrombin time and international normalized ratio, both being elevated. Intramural hematoma of jejunum was diagnosed by abdominal contrast-enhanced computed tomography. Conservative management was done, warfarin was stopped and vitamin K was administered. Patient received fresh frozen plasma and packed cell blood.

It is important to suspect warfarin toxicity in patients on the medication who come with such presentation to avoid surgical management, which could be catastrophic due to excessive bleeding. It is important for regular monitoring of coagulation profile of such patients and to reduce prescribing other medications that can interact with warfarin. It is worth noting that novel oral anticoagulants, such as dabigatran and rivaroxaban, are associated with fewer side effects and do not require close laboratory monitoring.

Acute intestinal obstruction is one of the most common surgical emergencies seen globally.

Intramural hematoma of intestine is usually seen due to blunt traumas, pancreatic disease, alcoholism, and hereditary clotting defects. A spontaneous intramural hematoma is rarely seen, and has been reported as one of the rare adverse effects due to anticoagulants therapy.

While surgical exploration is indicated in selected cases in acute settings, deranged coagulation profile may pose serious perioperative complications. Under these circumstances, a trial of expectant management, along with perioperative optimization, is warranted.

## Case Report

A 55-year-old male presented in casualty with acute pain in abdomen for the last 2 days and multiple episodes of vomiting for the last 1 day and obstipation for the last 1 day. On enquiry, patient gave history of multiple episodes of similar complaints over the last 3 months that resolved spontaneously. Patient was a known case of bronchial asthma and had been diagnosed with pulmonary thromboembolism 4 months back for which he was on maintenance on warfarin. The patient was also consuming oral terbinafine for tinea corporis infection. On examination, the patient had tachycardia. Abdominal examination revealed tenderness over periumbilical region. On digital rectal examination, the rectum was empty with no fecal staining of gloved finger.

**Fig. 1:**
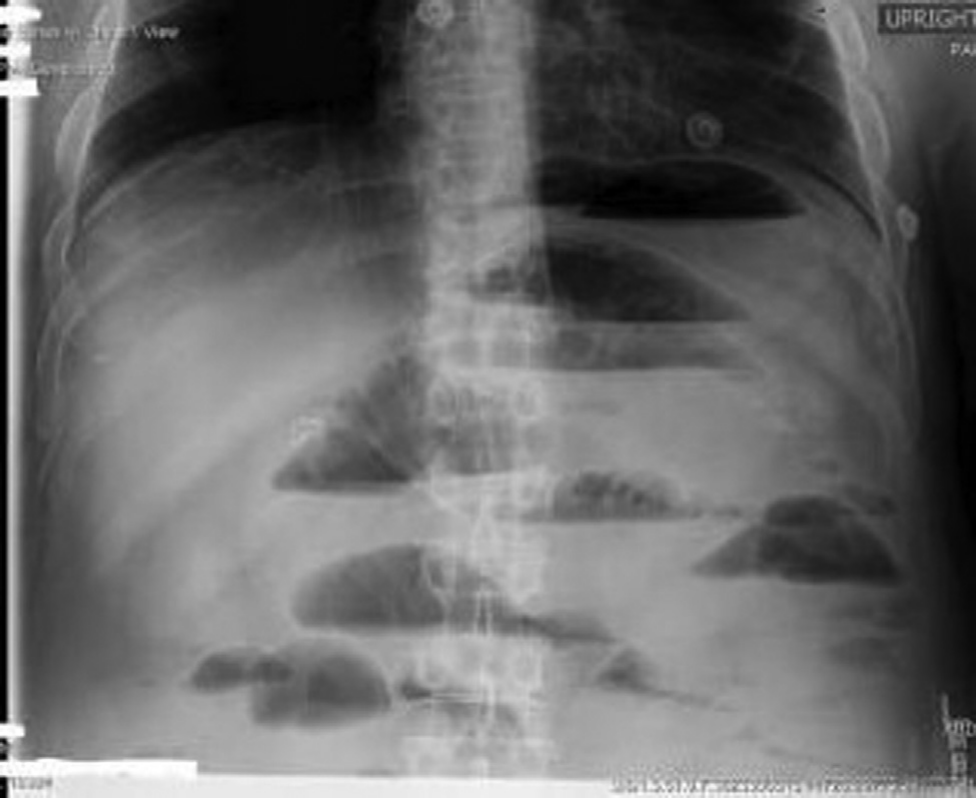
Erect abdominal radiograph showing multiple air fluid levels.

**Fig. 2:**
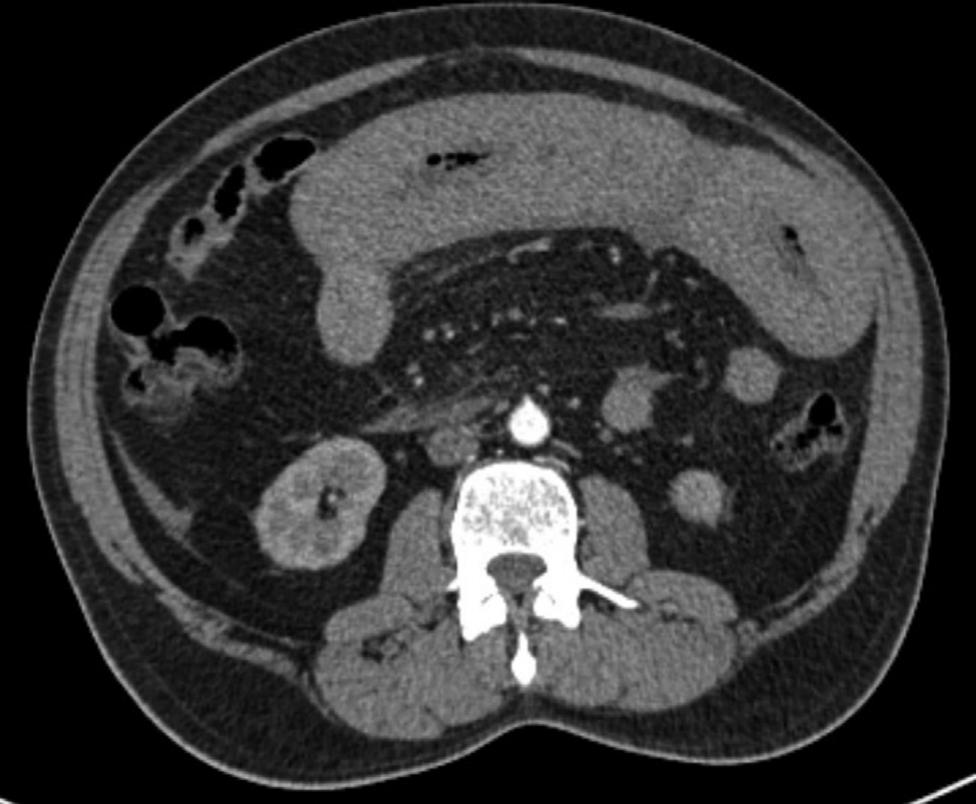
Contrast-enhanced computed tomographic scan of the abdomen showing intramural jejunal hematoma.

Erect abdominal radiograph showed multiple air fluid levels with dilated small bowel loops raising the diagnosis of acute small bowel obstruction ( Fig. 1 ). The contrast-enhanced computed tomography of abdomen and pelvis with oral and intravenous contrast showed long segment circumferential hyperdense wall thickening in proximal jejunal loops suggestive of intramural jejunal hematoma Fig. 2 .

Blood investigations revealed anemia with a hemoglobin of 6.2 g% along with mild leukocytosis. Coagulation profile was, however, grossly deranged, with prothrombin time being more than 100 seconds. Rest of the biochemical investigations were within normal limits.

The patient responded well to initial fluid resuscitation, and was therefore given a trial of expectant management with optimization for surgical procedure if and when needed. The patient was kept nil per oral, and warfarin was stopped. Measures were undertaken to correct the coagulation profile, with vitamin K injections and transfusion of fresh frozen plasma and packed red cells.

The patient passed stools on day 1 of admission in the ward following digital rectal stimulation. With the resolution of his symptoms and return of his bowel function, he was started on liquids by the morning of day 2 and soft diet by evening which were well tolerated. The patient was shifted to safer newer anticoagulant, dabigatran on discharge, and was followed up closely. Serial review of abdominal ultrasonography done in 2nd and 4th week post admission showed gradual reduction in size of hematoma, which was evident with reduced intestinal wall thickening. The hyperechoic lesion eventually became hypoechoic cystic in 2nd week followed by anechoic in 4th week. The patient did not complain of any similar episodes over 1 year of follow-up.

## Discussion

Warfarin is a vitamin K antagonist frequently used in thromboembolic conditions such as atrial fibrillation, prosthetic heart valve placement, and deep vein thrombosis. In cases especially where proper monitoring of coagulation profile, that is, prothrombin time and international normalized ratio (INR) are not done properly it commonly causes bleeding tendencies that are spontaneous and atraumatic in nature such as ecchymosis, subconjunctival hemorrhage, and gingival bleeding. 1 2

But formation of a spontaneous intramural hematoma of intestine has been a rare occurrence seen in approximately 1 out of every 2500 patients on anticoagulants a year. 2 3 It is also seen higher in patients who are on more than one anticoagulant or are on drugs that have higher chances if drug interactions. Jejunum is the most commonly affected part of the intestine followed by duodenum and ileum. Colon is rarely affected. 4 Most of these patients are asymptomatic, but when symptomatic, they have been seen to present with abdominal pain and vomiting, and some cases had history of hematemesis and melena. Our patient had a spontaneous intramural jejunal hematoma owing to deranged coagulation profile. The history of multiple similar episodes of symptoms over the past 3 months could be explained by formation and spontaneous absorption of the hematoma relieving the luminal compression.

A few decades ago, such a condition would have been managed by a surgical exploration followed by evacuation of hematoma or resection of the intestine. But now with the radiological development, such cases can be diagnosed early. Contrast-enhanced computed tomography (CT) abdomen has shown to be of a higher sensitivity and specificity in diagnosing this than an abdominal ultrasound. The typical features in the CT have found to be of circumferential thickening of the intestine, causing obstruction. Both barium enema and CT can show “picket fence” sign and “coiled spring” sign. 5

A higher value of INR is usually found in these cases. The patient may develop anemia due to loss of blood. In our case, the patient also had history of consumption of oral terbinafine for fungal infection. There may have been effect of terbinafine on warfarin metabolism as both are metabolized by liver, thus increasing chances of bleeding tendency. 6

Conservative management has been found to be the most effective in the few cases diagnosed all over the world. Cessation of the anticoagulant, vitamin K administration, and fresh frozen plasma are the initial steps. Resuscitation with intravenous fluids and correction of anemia with packed cell blood is required. Keeping the patient nil per oral gives rest to the bowel., enhancing the resolution of hematoma in the intestine. An expectant management both avoids the surgical stress on the patient and reduces the excessive bleeding that may occur on performing surgery. Surgical exploration that was commonly used before has now been reserved for cases where there was no response to conservative management or there was deterioration of patient, or obstruction associated with perforation. 4

Usually, cases of small bowel obstruction are managed surgically; however, here expectant management proved sufficient for the same. Also worth noting is that this is one of the rare cases of mechanical bowel obstruction that can be successfully managed with primary nonsurgical management.

Novel anticoagulants like dabigatran and rivaroxaban have the advantages compared with warfarin as they have minimal interactions with other drugs, rapid onset and offset of action, have predictable pharmacokinetics and dynamics and usually do not require laboratory monitoring. 7

## Conclusion

Intensive monitoring of patients on anticoagulants like warfarin is imperative to avoid potentially life-threatening complications and bleeding tendencies. Most of the patients consuming warfarin belong to the middle to older age groups wherein patients are on a cocktail of drugs. Inadvertent drug interactions in such cases pose a major threat.

Warfarin toxicity is the most common cause of spontaneous intramural small bowel hematoma. But due to the rarity of this condition, this particular etiology is often overlooked. Such instances point toward the importance of history taking. A proper diagnostic workup with radiological and blood investigations could avoid the disaster it would have caused on taking up the patient for surgical intervention.

Although rare, this condition is in a rising trend in the recent times due to the increase in the aging population being on a long-term anticoagulant therapy. The newer oral anticoagulants, such as dabigatran and rivaroxaban, which are more expensive than warfarin, have shown to have lesser adverse reactions and do not require close laboratory monitoring.
